# Impact of home-based management of malaria on health outcomes in Africa: a systematic review of the evidence

**DOI:** 10.1186/1475-2875-6-134

**Published:** 2007-10-08

**Authors:** Heidi Hopkins, Ambrose Talisuna, Christopher JM Whitty, Sarah G Staedke

**Affiliations:** 1 Department of Medicine, University of California, San Francisco, USA c/o MU-UCSF Malaria Research Collaboration, P.O. Box 7475, Kampala, Uganda; 2 Uganda Ministry of Health, P.O. Box 7272, Kampala, Uganda; 3 London School of Hygiene & Tropical Medicine, Keppel St, London WC1E 7HT, UK; 4 London School of Hygiene & Tropical Medicine, c/o MU-UCSF Malaria Research Collaboration, P.O. Box 7475, Kampala, Uganda

## Abstract

**Background:**

Home-based management of malaria (HMM) is promoted as a major strategy to improve prompt delivery of effective malaria treatment in Africa. HMM involves presumptively treating febrile children with pre-packaged antimalarial drugs distributed by members of the community. HMM has been implemented in several African countries, and artemisinin-based combination therapies (ACTs) will likely be introduced into these programmes on a wide scale.

**Case presentations:**

The published literature was searched for studies that evaluated the health impact of community- and home-based treatment for malaria in Africa. Criteria for inclusion were: 1) the intervention consisted of antimalarial treatment administered presumptively for febrile illness; 2) the treatment was administered by local community members who had no formal education in health care; 3) measured outcomes included specific health indicators such as malaria morbidity (incidence, severity, parasite rates) and/or mortality; and 4) the study was conducted in Africa. Of 1,069 potentially relevant publications identified, only six studies, carried out over 18 years, were identified as meeting inclusion criteria. Heterogeneity of the evaluations, including variability in study design, precluded meta-analysis.

**Discussion and evaluation:**

All trials evaluated presumptive treatment with chloroquine and were conducted in rural areas, and most were done in settings with seasonal malaria transmission. Conclusions regarding the impact of HMM on morbidity and mortality endpoints were mixed. Two studies showed no health impact, while another showed a decrease in malaria prevalence and incidence, but no impact on mortality. One study in Burkina Faso suggested that HMM decreased the proportion of severe malaria cases, while another study from the same country showed a decrease in the risk of progression to severe malaria. Of the four studies with mortality endpoints only one from Ethiopia showed a positive impact, with a reduction in the under-5 mortality rate of 40.6% (95% CI 29.2 – 50.6).

**Conclusion:**

Currently the evidence base for HMM in Africa, particularly regarding use of ACTs, is narrow and priorities for further research are discussed. To optimize treatment and maximize health benefits, drug regimens and delivery strategies in HMM programmes may need to be tailored to local conditions. Additional research could help guide programme development, policy decision-making, and implementation.

## Introduction

Malaria remains one of the major public health problems worldwide, and of the estimated 400 to 900 million episodes of fever occurring yearly in African children, probably about half are due to malaria, resulting in over one million deaths [1-3]. However, the proportion of deaths due to malaria varies widely with malaria transmission [4,5]. Prompt treatment with effective antimalarial therapy is essential and African leaders have committed to ensuring that 80% of malaria episodes are adequately treated within 24 hours of onset of symptoms by 2010 [6]. However, treatment of malaria is challenged by inadequate health-care infrastructure in many parts of Africa [7,8]. Health facilities are often resource-limited, and access to care may be limited by distance, fees, inadequate staffing, and lack of essential medicines [9-11]. The direct and especially indirect costs of seeking health care from formal facilities may be substantial, providing a major barrier for many households [12]. Thus, febrile illnesses are commonly treated at home, frequently with drugs purchased from shops [13]. It is estimated that fewer than 20% of children with malaria in endemic areas are treated in formal health-care settings [1].

To improve access to antimalarials, the World Health Organization (WHO) is promoting home-based management of malaria (HMM) as a major strategy for Africa [14]. HMM involves presumptively treating febrile children at or near home with antimalarial drugs distributed by trained members of the community [14,15]. Community distributors provide medications and educate primary caregivers about treatment of malaria, administration of antimalarial drugs, and recognition of severe illness. Emphasis on prompt treatment and distribution of pre-packaged antimalarials are strengths of the HMM strategy [16,17]. However, there are potential downsides to presumptive treatment at home. Use of antimalarials to treat all febrile episodes, even if administered correctly, may delay treatment of other illnesses [18]. In addition, unnecessary over-treatment with antimalarials could promote drug resistance [19,20] and is likely to have substantial economic consequences [21].

Recently, antimalarial treatment policies in Africa have undergone a major transition; most countries have adopted artemisinin combination therapies (ACTs) as first-line treatment for uncomplicated malaria [22]. Whether ACTs can be successfully incorporated into HMM and used safely and effectively at home is a critical question [23,24]. The cost of implementing ACTs in sub-Saharan Africa according to current prescribing practices has been estimated at US$ 1.6 – 3.4 billion per year, and the cost-effectiveness of deploying ACTs in HMM remains uncertain [21,23]. Quoted data supporting HMM policy are limited, and the impact of HMM on malaria-associated morbidity and mortality has not been fully established in most settings. The ideal regimen for use in HMM programmes is also not clear, and may vary by setting. This paper presents a review of published studies evaluating the health impact of community- and home-based treatment for malaria in Africa. The aim of this review is to summarize the current evidence base for HMM, and to identify areas where further research could guide implementation of HMM in Africa.

## Case presentations

A search of PubMed was conducted on 10 June 2007 using the keyword strategy in Table 1. All abstracts identified in the search were reviewed for potential relevance; if abstracts appeared to satisfy the stated criteria, the full text was obtained. Bibliographies of relevant publications and studies referred to in the gray literature (WHO and national reports) were also reviewed. Inclusion criteria for studies reviewed were as follows: 1) the intervention evaluated consisted of antimalarial treatment administered presumptively for febrile illness; 2) the treatment was administered by local community members who had no formal education in health care; 3) measured outcomes included specific health indicators such as malaria morbidity (incidence, severity) and/or mortality, and/or malariometric indices including parasite rates, hemoglobin or packed cell volume (PCV), and spleen rates; and 4) the study was conducted in Africa.

**Table 1:** Search strategy^a^

**Keyword Searches**
Home management of malaria (HMM);
Home-based management of malaria;
Home-based management of fever (HBMF);
Malaria AND community-based, community-directed, community participation, community care, community health volunteer, community health worker, community health aide, village health worker, village health volunteer, volunteer, volunteer health worker, lay health worker, birth attendant, midwife, traditional healer

^a ^PubMed search conducted 10 June 2007

**Figure 1:**
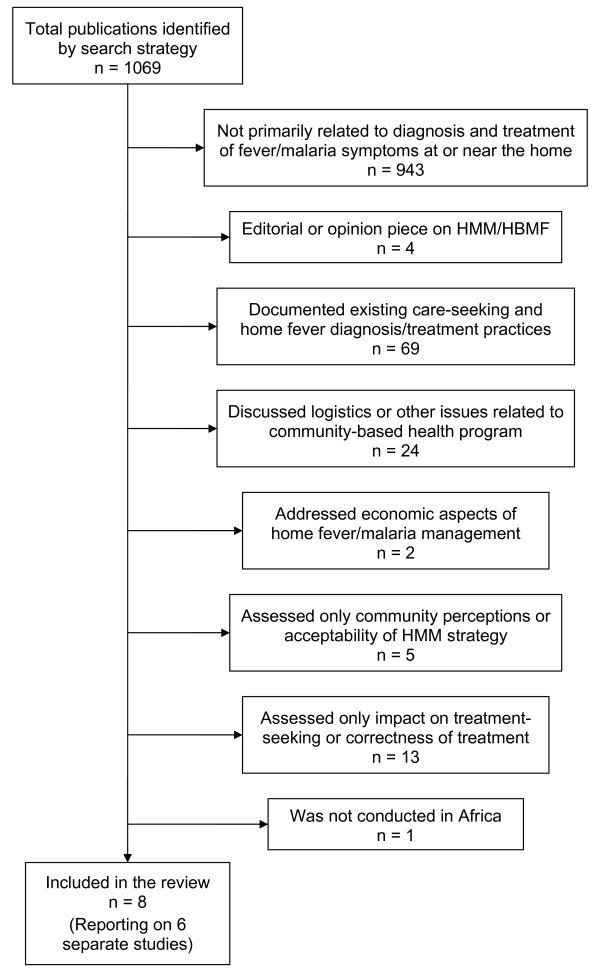
Categorization of published articles identified by the search strategy.

One thousand sixty-nine (1,069) potentially relevant articles were identified, most of which were not related to community-based diagnosis or treatment of malaria. Of the remainder, the following were excluded: a study that evaluated treatment in schools [25], as well as studies that measured only process indicators [26-28], assessed only changes in care-seeking and referral patterns [18,28], or evaluated only feasibility and acceptability [29-32]. A study from Niger, published in 1985, reported differences in malaria morbidity between villages with and without village health workers who provided both chemoprophylaxis and presumptive treatment with chloroquine (CQ), but there were no data distinguishing the effects of treatment from those of chemoprophylaxis [33]. Only one study, also published in 1985, was excluded on the basis of being conducted outside Africa; this study reported on the health impact of a village-based programme for presumptive antimalarial treatment of fever in Papua New Guinea [34]. Six studies that met all inclusion criteria were identified (Figure 1).

**Figure 2:**
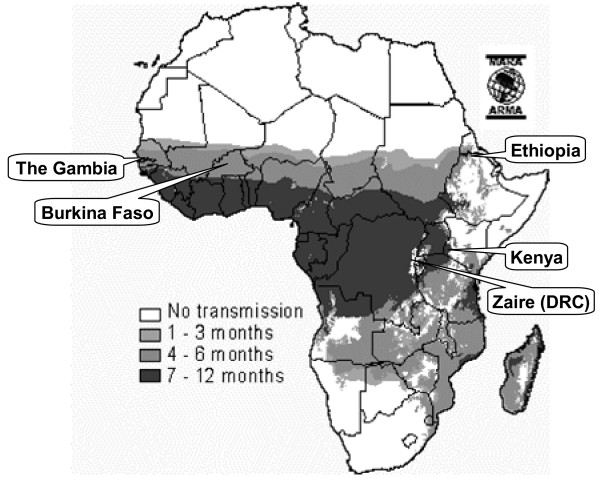
Sites of published studies on the health impact of home- and community-based treatment for malaria in Africa. Two studies were conducted in Burkina Faso. The map, adapted from MARA: Mapping Malaria Risk in Africa [69], shows seasonality of malaria transmission in months per year, as in the key.

Six studies that involved presumptive administration of antimalarial treatment at or near the household level and that met the search criteria were identified (Table 2 and Figure 2) [35-42]. One project in Kenya [35,36] and another in The Gambia [37,38] were described in multiple papers.

**Table 2:** Characteristics of published studies of home- and community-based treatment for malaria in Africa^a^

**Location**	**Epidemiology**	**Drug distribution**	**Incentive**	**Outcomes measured**	**Results**
Kenya 1981–83 (Spencer et al, 1987a, 1987b)	• Rural • Hyper- to holo-endemic	• CHWs provided presumptive CQ treatment for free	• Volunteer CHWs supported by the village	• Overall and malaria-specific mortality • Birth and fertility rates • Parasite rates	No obvious effect of providing CQ for treatment of malaria on mortality, fertility, or parasite rates

The Gambia 1982–87 (Greenwood et al, 1988; Menon et al, 1990)	• Rural • Seasonal transmission	• CHWs sold CQ for presumptive treatment	• Volunteer CHWs supported by the village	• Overall and malaria-related mortality • Frequency of clinical malaria • Packed cell volume, parasite rates, splenomegaly	Treatment alone had no significant effect on morbidity and mortality from malaria

Zaire (DRC) 1985–87 (Delacollette et al, 1996)	• Rural • Meso-endemic • Continuous transmission with seasonal fluctuations	• CHWs sold CQ at cost for presumptive treatment	• CHWs received "symbolic monetary reward"	• Malaria morbidity and mortality • Parasitological indices • Proportion of fever episodes receiving antimalarial treatment, proportion receiving treatment at home, and source of treatment	No impact on malaria mortality, but two-fold reduction in malaria prevalence and incidence

Burkina Faso 1994–95 (Pagnoni et al, 1997)	• Rural • Seasonal transmission	• Mothers trained to recognize illness and make decision to treat • CHWs sold pre-packaged CQ for presumptive treatment	• CHWs kept 0.6 US cents for each package sold	• Proportion of under-5 malaria cases recorded as severe in health centres • Mothers' care-seeking practices • Availability and use of drugs at peripheral level, community awareness of educational messages	The proportion of severe cases decreased in the first year of the program; in the second year, the proportion decreased only in health facilities with drug coverage ≥50%

Ethiopia 1996–98 (Kidane and Morrow, 2000)	• Rural • Seasonal transmission	• Mother coordinators provided presumptive CQ treatment for free	• None mentioned	• Malaria-related mortality in children under age 5 years	Intervention associated with 40.6% reduction in overall under-5 mortality (95% CI 29.2–50.6, p < 0.003)

Burkina Faso 1998–99 (Sirima et al, 2003)	• Rural • Hyperendemic • Seasonal transmission	• Mothers trained to recognize illness and make decision to treat • CHWs sold pre-packaged CQ for presumptive treatment	• Drugs sold with 10% incentive margin for CHW • Incentive provided to some drug store managers	• Proportion of malaria cases progressing to severe (as reported by mothers in annual cross-sectional surveys) • Proportion of cases receiving correct dose of CQ	Risk of progression to severe malaria lower in children treated promptly with pre-packaged CQ (5%) than not (11%) (RR 0.47, 95% CI 0.37–0.60, p < 0.0001)

^a^CHW = community health worker; CQ = chloroquine; RR = risk ratio

### Kenya 1981–1983

This community-based project was conducted in a hyper- to holo-endemic area near Lake Victoria [35,36,43,44]. All households were registered, and the community was divided into three areas, two interventional, and one control. Community health workers (CHWs) were trained to give CQ free of charge "to every person who came for treatment saying they had malaria," and to refer ill patients. Each household was revisited at six to nine month intervals and information on births, deaths, and migration was recorded. Biannual surveys were also conducted in randomly selected villages to assess parasitemia and antimalarial antibodies.

Despite high utilization, presumptive treatment with CQ was found to have no impact on malaria-specific or overall mortality. Further, there was no change in parasite prevalence or serologic markers. The authors attributed the lack of effect to the high level of presumptive treatment with CQ in the community prior to the onset of the programme.

### The Gambia 1982–1987

A community-based programme in an area with seasonal transmission was conducted in 1982–85 [37], with follow-up assessments in 1986–87 [38]. Forty-one villages were stratified according to population size. Larger villages were selected to participate in a national primary health care scheme, which began in 1983, while the smaller villages served as a control group. The CHWs received health-care training, including presumptive treatment of malaria with CQ, over a 6-week period. An initial stock of basic medicines was provided, and CHWs were expected to purchase new supplies using funds from drug sales. A group of CHWs also distributed either pyrimethamine/dapsone chemoprophylaxis or placebo every two weeks to children aged 3–59 months, randomly allocating children by household. The study compared two strategies for malaria control: presumptive treatment of malaria with CQ alone or combined with pyrimethamine/dapsone prophylaxis.

In the initial evaluation, presumptive treatment of fever with CQ was found to have no impact on morbidity or mortality. However, in children aged 1–4 years, pyrimethamine/dapsone prophylaxis in addition to CQ treatment led to a significant decrease in mortality and incidence of clinical malaria, a fall in spleen and parasite rates, and an increase in packed cell volume. Similar findings were observed in the follow-up evaluation, conducted 3–4 years later. The authors noted that CQ resistance, first described in The Gambia one year after this study was completed, was unlikely to account for the lack of effect of presumptive CQ treatment. Instead, the ineffectiveness of the programme was attributed to the volunteer status of the CHWs and their limited availability.

### Zaire (Democratic Republic of Congo) 1985–1987

This study was conducted in a meso-endemic area on the western shore of Lake Kivu [39]. Two regions were chosen, one for the intervention, and the other for the control. "Literate volunteers" were selected by the community to serve as CHWs, and were trained to administer "timely" presumptive treatment with CQ.

The intervention was associated with a decrease in malaria morbidity: a two-fold reduction in mean malaria prevalence and incidence, and a five- to six-fold decrease in parasitological indices were observed. However, there was no difference in malaria mortality, and 24% of fever cases remained untreated at the end of the observation period. The authors attributed the lack of mortality impact to the relatively small sample size (total population 28,000), and limitations of verbal autopsies for determining malaria-related deaths. They also suggested improved availability of CQ, rather than the specific mechanism of its delivery, may have accounted for some of the observed morbidity benefit. In addition, a number of problems concerning the relationship of the CHWs to the health care system and the community were observed, leading the authors to question the sustainability of a community-based programme with dedicated CHWs. "The non-comprehensiveness of the CHWs' care and their ambiguous position in the health care system created problems that compromise the sustainability of the intervention."

### Burkina Faso 1994–1995

This study was carried out in one province in north-western Burkina Faso with seasonal malaria transmission [40]. The primary aim of the study was to assess the impact of community-based provision of prompt antimalarial treatment on the risk of progression to severe disease. The intervention involved teaching mothers to recognize malaria episodes and providing pre-packaged CQ through CHWs. A "core group" of mothers and CHWs received training from local nurses in the diagnosis and treatment of uncomplicated malaria, and were expected to train other mothers in the village. Emphasis was placed on entrusting mothers with the decision to treat their children. Pre-packaged CQ was supplied to health dispensaries, and the CHWs sold full treatments under a cost-recovery scheme. Pre- and post- intervention data were collected to allow for historical comparisons. National Health Information System (NHIS) data on the number of patients with "uncomplicated" or "severe" malaria presenting at dispensaries were used to evaluate the impact of the intervention. Of note, the intervention was transferred from the national to provincial level midway through the study, which affected the distribution of the drugs and supervision of the programme.

A decrease in the proportion of severe malaria cases recorded in health centre log books was noted during 1994, as compared to the four preceding years. In 1994, 258 of 6725 (3.8%) malaria cases were documented as severe, compared to 704 of 14314 (4.9%) of cases in 1990–1993 (reported p = 0.0005). In 1995, the results were stratified by drug availability at the health centres. In health centres with a drug coverage index of ≥ 50% (47% of dispensaries) the proportion of severe cases was lower than in previous years. However, in facilities with poor drug availability, the proportion of severe cases was higher than in 1990–93. The authors comment on several limitations of the study including the use of NHIS data for the primary endpoint and comparison to historical controls. Dispensary records of severe malaria typically correspond with cerebral malaria only, limiting detection of other forms of complicated malaria. In addition, nurses recording the malaria cases at the health facilities were also involved in implementing the programme, which may have influenced recording of malaria cases. However, the authors concluded that the results of this study confirmed the feasibility and affordability of community-based presumptive treatment, and suggested a morbidity benefit. Empowering mothers as decision-makers for diagnosis and treatment, and the cost-recovery scheme of the programme, were considered to be strengths of the intervention.

### Ethiopia 1996–1998

This trial examined the effect of "teaching mothers to promptly provide antimalarials to their sick children at home," as compared to health facility-based treatment, on under-5 mortality [41]. The study was conducted in two districts of Tigray, a region with mostly seasonal malaria. The 24 tabias (clusters of villages) with the highest malaria morbidity were paired by their under-5 childhood mortality rates. One tabia from each pair was randomly assigned to the intervention, the other to the control. "Mother coordinators" in all tabias were trained to keep records of local births and deaths and to refer sick children, and were responsible for tracking drug supplies at the local health centre. In the intervention tabias, mother coordinators were additionally trained to teach other local mothers to recognize symptoms of malaria, and to give prompt CQ treatment. CQ was supplied to the mother coordinators in the intervention group for distribution to all households, and the mothers were responsible for replenishing the drug supply.

This widely-cited study found a reduction in the under-5 mortality rate of 40.6% (95% CI 29.2 – 50.6). In the intervention group, 190 of 6383 children died (29.8 per 1000), compared to 366 of 7294 (50.2 per 1000) in the control group (p < 0.003). The authors did note that the Tigray region was involved in a civil war from 1974 until 1991; after years of war and isolation, local characteristics were seen as possible reasons for the success of the programme. The authors emphasize that "it is important to recognize the special biological and sociopolitical factors of the Tigray study that may limit applicability in other parts of Africa such as the presence of chloroquine-sensitive falciparum malaria, a disciplined population accustomed to coping for themselves, strong community solidarity, and no alternative income opportunities for the mother coordinators."

### Burkina Faso 1998–1999

This observational study was conducted in south-eastern Burkina Faso, an area with hyper-endemic seasonal malaria [42]. A "core group of opinion leaders," mostly older mothers, and CHWs were trained in diagnosis of malaria, referral of cases, and treatment with pre-packaged CQ and aspirin. Health centre drug store managers were trained to package age-specific doses for sale to CHWs, who in turn sold the drugs to local mothers, at a price allowing for full cost-recovery of the drugs plus a 10% incentive margin. Outcomes for children treated promptly (within 24 hours) with pre-packaged antimalarials were compared to those who did not receive such treatment. The outcome of "severe malaria" was defined as an episode of fever followed by convulsions or loss of consciousness, as reported by mothers.

Of 1806 children receiving prompt treatment with pre-packaged CQ, 93 (5%) progressed to severe malaria, compared to 153 of 1396 (11%) of children who did not receive this treatment (risk ratio 0.47, 95% CI 0.37 – 0.60, p < 0.0001). The authors address the possible impact of non-differential misclassification, recall bias, lack of parasitological confirmation of disease, and confounding factors on their findings, concluding that these were unlikely to affect their positive results. The authors noted that this study only provides evidence on risk of progression to cerebral malaria, not other clinical forms of malaria, including severe anemia.

## Discussion and evaluation

Prompt treatment with effective antimalarials is a cornerstone of malaria control in Africa. HMM has the potential to improve treatment delivery and decrease malaria-associated morbidity and mortality. However, the HMM strategy is a major undertaking, and its implementation should be based on sound evidence of public health benefit. To assess the current evidence base for HMM, a review was conducted of published studies that evaluated the health impact of community- and home-based treatment for malaria in Africa. The six studies identified were conducted in diverse epidemiological settings in five countries over a span of 18 years. Heterogeneity of the evaluations, including variability in study design, precluded meta-analysis. All studies were conducted in rural areas, and most were done in settings with seasonal malaria transmission. Of the two trials conducted in perennial transmission areas, one in Kenya showed no obvious effect of the intervention [35,36], while the other in the Democratic Republic of Congo showed a decrease in malaria morbidity but no impact on mortality [39]. All studies evaluated presumptive treatment with CQ, and only two studies (both in Burkina Faso) reported using pre-packaged drugs, a key element of the HMM strategy; critically, no data are available on use of ACTs or other alternative therapies [45]. Of the four studies that included mortality endpoints, only one showed a benefit [41]. Data showing an impact of community-based programmes on malaria morbidity are also relatively sparse with only one study showing a decrease in malaria prevalence and incidence [39], and one demonstrating a decreased risk of progression to severe (essentially cerebral) malaria [42]. Thus, current evidence demonstrating the health benefit of home- and community-based presumptive treatment of fever with antimalarials is limited, and does not necessarily support widespread implementation of HMM.

The WHO has advocated scaling up home-based programmes in malaria-endemic countries, supported by the positive results of the two most recent studies [14,41,42]. As of 2004, three countries in Africa (Eritrea, Ethiopia and Uganda) were implementing the full HMM strategy with a non-ACT regimen, while other countries were incorporating some components of the strategy [15]. In Uganda, HMM was initiated with pre-packaged CQ + sulfadoxine-pyrimethamine (Homapak), but artemether-lumefantrine (AL) has recently been introduced in the HMM programme in northern districts, and widespread introduction of AL is expected in the future. Although there are plans to evaluate use of AL in HMM in Uganda, and a community-based trial of AL is currently on-going in Burkina Faso (Sirima, personal communication), currently there are no published data on use of ACTs in HMM programmes.

Provision of ACTs in HMM could have substantial economic and public health implications. Implementation of home- or community-based programmes based on non-ACT regimens (CQ or CQ plus sulfadoxine-pyrimethamine) have been shown to be relatively affordable [40,46], and a cost-effectiveness model found that provider training, community education and pre-packaging of CQ compared favorably with other malaria control measures [47]. The system costs of implementing HMM are expected to be similar regardless of which regimen is used; however, the cost of deploying ACTs in HMM will certainly be higher than that of CQ. Although the effectiveness of HMM with ACTs is likely to be greater than with CQ, this has not been demonstrated. In addition, further evaluation of the cost-effectiveness of HMM, particularly using ACTs, would be instructive. Deployment of ACTs just within the public health sector already poses a significant challenge to many countries due both to cost and to limitations in the global supplies of artemisinin compounds [48-50], and the costs and benefits of further deployment of ACTs in HMM programmes should be assessed.

Presumptive treatment of all childhood fevers in Africa would greatly increase ACT demand and drug pressure, even if just a proportion of fevers were actually treated. A number of studies suggest that increased antimalarial use speeds the emergence and spread of parasite resistance [20,51-53]. Instead of deploying a single ACT regimen as first-line treatment in both health facilities and HMM, use of different regimens in different settings may be an alternative approach. Early modeling work suggests that using more than one ACT in a region, e.g. administering different ACTs in facilities and in HMM, may slow development of resistance, although this theory requires further investigation [54]. Poor patient adherence to treatment is also likely to contribute to the development of parasite resistance, through exposure of parasites to subtherapeutic drug levels [55,56]. Use of pre-packaged drugs, as promoted for HMM, has been shown to improve adherence [16,17] which may deter parasite resistance. The impact of widespread presumptive use of ACTs in HMM programmes on the development of drug resistance requires further study.

Considering the available literature, how can future research address the gaps in evidence, especially in the era of ACTs? Evaluation of the impact of community-based treatment on malaria-associated morbidity and mortality in different epidemiologic settings, particularly areas with perennial malaria transmission, would be useful to help support wide-scale implementation of HMM. Additional questions exist regarding the optimal regimen for HMM, method of drug distribution and maintenance of supplies, use of incentives, programme design in different social settings (e.g. rural vs urban), safety risks, sustainability, and cost-effectiveness. A recent WHO document provides a detailed review of the implementation processes of HMM studies conducted in Burkina Faso, Ghana, Nigeria and Uganda, identifying "lessons learned" and challenges for future programme planning [45]. Observations summarized in this document can help to identify opportunities to improve existing HMM programmes and directions for further research. In countries that plan to implement HMM with ACTs, research could be closely linked to implementation, for example by randomizing communities to HMM with ACTs in a phased design with a robust impact assessment data collection system. Further, it will be important to evaluate the impact of different models of delivery of ACTs. For example, what is the added value of HMM with ACTs in a setting where formal health care is strengthened, minimizing medicine stock-outs and motivating staff?

The question of payment of CHWs is an important issue: financial compensation may provide incentive for CHWs to provide consistent service, but payment may also disrupt existing social patterns of reciprocity, and contribute unnecessarily to the cost of the programme [57]. The WHO recommends that CHW incentives and remuneration should be agreed upon with the communities where programmes are implemented [45]. The impact of CHWs, and different compensation approaches, on local socioeconomic structures may be an interesting area for further research. An additional challenge for community-based programmes is the shift in social structures and malaria epidemiology brought on by rapid urbanization in sub-Saharan Africa [58,59]. Community health interventions in rural areas may be more successful than in urban areas with looser social ties. This effect has already been seen with community-directed treatment for lymphatic filariasis in Ghana, where the programme in urban areas was affected by the "lack of closely knit community structure and ... intense migration" [60]. Consideration of social networks in urban areas will be necessary if successful community-based health programmes are to be implemented in cities, where an increasingly large percentage of the African population lives.

Use of diagnostics to target therapy versus presumptive treatment of fever is another important question. Empiric treatment of fever with antimalarials is widely advocated and practiced in Africa, although presumptive diagnosis has been demonstrated in many settings to be highly inaccurate [61-64]. Field evaluations of commercially available rapid diagnostic tests (RDTs) for malaria have shown that personnel with no formal training are able to satisfactorily prepare and interpret these tests [65,66]. RDTs may prove useful in targeting treatment to patients most likely to be suffering from malaria, and their adoption in African HMM programmes should be studied. However, the successful use of RDTs will require a significant cultural shift in the way health workers deal with negative test results [67,68].

## Conclusion

Ideally, all children with malaria in Africa should be treated promptly with effective antimalarials. Presumptive treatment of febrile children with pre-packaged antimalarials in HMM programmes is likely to increase delivery of effective drugs, and improve the timing, adherence, and dosing of treatment. Results from evaluations of community acceptability and feasibility are encouraging, but further study of health outcomes, including the impact on morbidity and mortality, will provide stronger evidence to support sustained implementation of community-based interventions. Introduction of ACTs into HMM will require substantial investment, and may divert funds from other public health programmes and investment in health facilities. Malaria epidemiology, drug resistance patterns, health infrastructure, and societal structures vary widely across Africa. The approach to drug delivery and treatment will likely need to be tailored to local conditions. Further evaluations of HMM and community-based delivery of antimalarials will help to optimize treatment strategies and maximize health benefits.

## Authors' contributions

HH and SGS independently performed the literature search, reviewed all articles cited and drafted the manuscript. AT and CJMW confirmed the findings and conclusions, and provided additional commentary and perspective. All authors contributed to and approved the final manuscript.
